# Temporal profile of intranasal oxytocin in the human autonomic nervous system at rest: An electrocardiography and pupillometry study

**DOI:** 10.1177/02698811231158233

**Published:** 2023-03-09

**Authors:** Gonçalo Cosme, Patrícia Arriaga, Pedro J. Rosa, Mitul A. Mehta, Diana Prata

**Affiliations:** 1 Instituto de Biofísica e Engenharia Biomédica, Faculdade de Ciências, Universidade de Lisboa, Lisboa, Portugal; 2 Instituto Universitário de Lisboa (ISCTE-IUL), CIS-IUL, Lisboa, Portugal; 3 HEI-LAB: Human-Environment Interaction Lab/Universidade Lusófona de Humanidades e Tecnologias, Lisboa, Portugal; 4 ISMAT, Transdisciplinary Research Center (ISHIP), Portimão, Portugal; 5 Institute of Psychiatry, Psychology and Neuroscience, King’s College London, London, UK

**Keywords:** Electrocardiography, neuropeptide, pharmacodynamics, pupil unrest, resting state

## Abstract

**Background:**

Human social behavior is modulated by oxytocin (OT). Intranasal administration of OT (IN-OT) is a noninvasive route shown to elicit changes in the autonomic nervous system (ANS) activity; however, IN-OT’s effect on the temporal profile of ANS activity at rest is yet to be described.

**Aims:**

We aimed to describe the temporal profile of IN-OT at six 10-min time windows from 15- to 100-min post-administration in 20 male participants at rest while continuously recording their pupillary in an eyes-open condition and cardiac activity in eyes-open and eyes-closed conditions.

**Methods:**

We used a double-blind, placebo-controlled, within-subjects design study where we extracted two proxies of parasympathetic nervous system (PNS) activity: high-frequency heart rate variability (HF-HRV) and pupillary unrest index (PUI); and a proxy of sympathetic nervous system activity: sample entropy of the pupillary unrest.

**Results:**

In the eyes-open condition, we found an effect of IN-OT on the proxies of PNS activity: decreased PUI in the three-time windows post-administration spanning 65–100 min, and as an exploratory finding, an increased HF-HRV in the 80–85 min time window.

**Conclusions:**

We suggest there is a role of OT in PNS regulation that may be consistent with OT’s currently theorized role in the facilitation of alertness and approach behavior.

## Introduction

Oxytocin (OT) has increasingly gathered the interest of cognitive neuroscientists since it was shown to be implicated in social cognition and behavior in humans (Erdozain and Peñagarikano, 2020) and potentially in the elusive pathophysiology of social symptoms in psychiatric disorders such as autism spectrum disorder (Huang et al., 2021; Zhao et al., 2022), schizophrenia (Liu et al., 2019), and borderline personality disorder (Jawad et al., 2021). Highly promising early studies (Averbeck et al., 2012) gave hope that pharmacological administration of OT may mitigate social behavioral deficits in these conditions (Huang et al., 2021; Shilling and Feifel, 2016). However, there is, thus far, inconsistency in findings, variability in the methods and in the outcomes and response biomarkers measured (Winterton et al., 2021), with insufficient metanalytical evidence of improvement in clinical populations (Huang et al., 2021; Sabe et al., 2021). Intranasal OT (IN-OT) is, by far, the most frequent route of OT administration in human neuroscience studies, and we have recently summarized these studies (Zelenina et al., 2022). Overall, IN-OT’s temporal profile (i.e., across a typical neuroscience experimental session time) at rest has been characterized by OT measurement in peripheral fluids (i.e., blood plasma, saliva and urine) and central nervous system OT measurements (i.e., in cerebral spinal fluid) or activity (via blood oxygen level-dependent activation using magnetic resonance imaging (MRI), and, by us, microstates using electroencephalography (EEG) (Zelenina et al., 2022). However, to the best of our knowledge, the temporal profile of IN-OT is still unexamined in the peripheral nervous system at rest. Such examination is of crucial importance for neuroscience studies’ design and interpretation because the autonomic nervous system (ANS) activity is associated with a myriad of social cognitive processes (Jáuregui et al., 2011; Quintana et al., 2012), as we have recently shown for cognitive empathy (Cosme et al., 2021, 2022). The ANS is also more easily accessible than the central nervous system in humans. Besides, such knowledge would be useful for developing biomarkers predictive of IN-OT treatment response (Erdozain and Peñagarikano, 2020; Quintana et al., 2021; Winterton et al., 2021) in preparation for clinical trials.

The effects of OT on social cognition have been linked to both central and peripherally measured nervous system activity and integrated into hypotheses such as the social salience hypothesis (Shamay-Tsoory and Abu-Akel, 2016), the general approach-avoidance hypothesis (Harari-Dahan and Bernstein, 2014) and the allostatic hypothesis (Quintana and Guastella, 2020). Heart rate variability (HRV), for example, has been implicated in social cognition (Park and Thayer, 2014), such that an increase in HRV has been associated with motivation towards social engagement (Beffara et al., 2016). Contrarily, a reduced HRV has been found in subjects with difficulties in emotion regulation (Mather and Thayer, 2018), in autism spectrum disorders (Lory et al., 2020) and first episode psychosis (Cacciotti-Saija et al., 2018) and, while at rest, may impair emotion regulation (Park and Thayer, 2014). What is not known, currently, is how the effects of OT on HRV, and other peripheric measures, at rest, integrate into OT’s cognitive hypotheses. As a first step, in this study, we aimed to develop an understanding of the effects of OT on ANS activity at rest.

OT is produced in the paraventricular, supraoptic, and accessory magnocellular nuclei of the hypothalamus (Meyer-Lindenberg et al., 2011) with direct projections to the dorsal brain stem, which regulates cardiovascular activity (Gutkowska et al., 2014), and the amygdala, which regulates ANS response patterns, particularly heart rate (Yang et al., 2007), the heart being replete with OT receptors (Gutkowska et al., 2014). Altogether, cardiac indices are suitable to assess IN-OT effects on ANS activity, particularly the parasympathetic branch. Research has also shown that increased OT receptor gene methylation (i.e., silencing) is associated with decreased parasympathetic nervous system (PNS) activity at rest via measurement of this system’s well-known positive proxy (Shaffer and Ginsberg, 2017): high-frequency heart rate variability (HF-HRV) (Lancaster et al., 2018). High HF-HRV has been associated with increased accuracy in identifying others’ positive states, which may encourage longer and more successful social relationships, and approach behaviors (Lischke et al., 2017). At rest, increased HF-HRV has also been found to predict cooperative behavior (Beffara et al., 2016) which, in turn, has been associated with increased OT function (Dreu, 2012; Rilling et al., 2012).

Yet, the characterization of IN-OT’s impact on the ANS function is still unclear, both during cognitive tasks and at rest. So far, IN-OT’s effects on each branch of the ANS have been inconsistent. During tasks, it has been found to (1) increase PNS activity (specifically, indexed by increased HF-HRV) in a facial emotion recognition task, albeit with no effect on the sympathetic nervous system (SNS) measured by electrodermal activity (Gamer and Büchel, 2012); (2) decrease PNS activity (specifically, indexed by lowered HF-HRV) while also increasing the low frequency (LF-HRV), whose meaning is still unclear, during a mental arithmetic task (Tracy et al., 2018); and (3) have no effect on PNS (specifically via HF-HRV) but increased SNS activity indexed by decreased pre-ejection period (PEP), during a social stress task (Kubzansky et al., 2012). However, the purity of the PEP as a proxy for the SNS has since been questioned given it has been associated with many other cardiovascular factors (Krohova et al., 2017). During rest, the findings also remain inconsistent by showing that IN-OT (1) coactivates both PNS and SNS (specifically, via decreasing the nonlinear HRV parameter short-term Detrended Fluctuation Analysis (DFAα1), which has been negatively associated with activation of both branches (Tulppo et al., 2005)) during a 10-min eyes-closed seated rest (Kemp et al., 2012); (2) increases PNS activity (via heightening HF-HRV) (Kemp et al., 2012), and (only) immediately after a social stress task in another study (Kubzansky et al., 2012); (3) decreases PNS activity (specifically by decreasing the root mean square of successive differences (RMSSD)) but only in females with positive childhood rearing experiences (Schoormans et al., 2020); and (4) has no influence on resting-state ANS activity (measured by HF-HRV and LF-HRV, the interval between successive R peaks (RRI) and RMSSD) (Tracy et al., 2018). All the abovementioned resting-state studies used the commonly applied 24 IU of IN-OT and a single time window, with variable lengths, albeit overlapping at 40- to 45-min post-administration. To our knowledge, only one study attempted to characterize the temporal profile, of IN-OT on HRV, but it was task-based, which we discuss later on (Norman et al., 2011).

Pupillary oscillations also reflect ANS activity; however, it has not yet been used to help characterize the effects of IN-OT at rest. The pupil’s constriction is controlled by the sphincter muscle, innervated by the PNS, and its dilation is controlled by the dilator muscle, innervated by the SNS (Mathôt, 2018), thus, the overall pupil size is modulated by the interplay of both branches of the ANS. At rest, the pupil naturally dilates and constricts in a spasmic and rhythmic fashion (Lüdtke et al., 1998). The pupillary unrest index (PUI) (Lüdtke et al., 1998; Schumann et al., 2020) is a measure of these fluctuations’ occurrence, representing the deviation in pupil dilation at low frequencies, and has been positively associated with PNS activity (using cardiac indices as proxies such as RMSSD) and, in specific, sleepiness, but negatively with alertness (Lüdtke et al., 1998; Schumann et al., 2020), and it varies with the time of day (Danker-Hopfe et al., 2001). On the other hand, sample entropy (SampEn) is a measure of the pupillary unrest’s complexity (Richman and Moorman, 2000) and has been positively associated with SNS activity (as measured by skin conductance indices) (Schumann et al., 2020). In terms of task-based research, two studies have reported increases in emotional faces stimulus-induced mean pupil dilation at 40-min post-administration, one using 24 IU and another 40 IU (Leknes et al., 2013; Prehn et al., 2013). A third study found that 8 IU of IN-OT, when administered via a Breath Powered nasal device, elicits lower facial stimuli-induced pupil dilation compared to 24 IU or placebo (Mahmoud et al., 2018), which may be explained by the dose-effect inverted-U-shaped curve previously observed for IN-OT (Borland et al., 2019).

In sum, the temporal profile of IN-OT’s effect on the ANS at rest remains to be examined, and its impact at a commonly reported time window of assessments of around 40 min is not known. In the present study, we aimed to assess the effect of 24 IU IN-OT on ANS activity at rest, across a large neuroscience experimental session duration, in healthy males, with a double-blind, randomized placebo-controlled cross-over design, recording their pupillary and cardiac activity at one baseline time window pre-administration and at six time windows post-administration (from 15 to 100 min). We examined the effect of IN-OT on proxies of PNS and SNS activity, in each time window, using electrocardiography (ECG) and pupillometry signals: two positive proxies of PNS activity (HF-HRV and PUI) (Schumann et al., 2020; Shaffer and Ginsberg, 2017) and one of SNS activity (SampEn) (Schumann et al., 2020). We specifically chose HF-HRV, a frequency measure of PNS activity, in contrast to time-domain ones (e.g., RMSSD), for comparability with previous IN-OT studies (Kemp et al., 2012; Kubzansky et al., 2012; Norman et al., 2011; Schoormans et al., 2020; Tracy et al., 2018). (However, for completeness, we report other available indexes in Supplemental Material—as explained in the Methods.) Our primary hypothesis was that IN-OT would coactivate the PNS and the SNS as reflected in an increased heart rate’s HF-HRV (Kemp et al., 2012; Kubzansky et al., 2012) which is considered a robust positive proxy of PNS activity. Aiming at providing converging evidence, we also used the pupil size’s PUI and SampEn as secondary outcomes despite being more indirect (Schumann et al., 2020) and less studied proxies of PNS and SNS, respectively. Nonetheless, we predicted that they would increase with IN-OT. These predictions are based on previous (and abovementioned) two studies’ consistent reports (one at rest and the other at rest following a social stress task), with one exception (Tracy et al., 2018) of IN-OT increasing PNS and SNS activity measured by nonlinear measures of HRV, via HF-HRV and DFAα1 (Kemp et al., 2012; Kubzansky et al., 2012), while no previous pupillometry findings are available. The aim is for our findings to assist in (1) future study design regarding the selection of the optimal IN-OT neuroscientific experimental sessions length, (2) comparability between previous and future IN-OT findings using different time windows and data modalities, (3) assessing the potential usefulness of these ANS markers as IN-OT treatment response monitoring tools, and (4) advancing our understanding of the role of OT in human cognition and behavior.

## Materials and methods

### Participants

We recruited 20 young (*M*_age_ = 27.4; *SD*_age_ = 3.88, age range = 22–34), healthy, male, Portuguese adults, through mailouts and pamphlets in the university community and online social networks. All participants were included in the analysis. Exclusion criteria were self-reported history of endocrinological, cardiovascular, or neurological disorders; substance abuse, blocked nose; consumption of cannabis within 2 weeks prior to data collection; alcohol consumption, drugs or any medication within 24 h prior; caffeine consumption or heavy physical exercise or sexual activity on the experiment day, or tobacco smoking less than 2 h prior to the experimental session. All participants gave their written informed consent and received financial compensation for their time. The study was approved by the Ethics Committee of the Lisbon Medical Academic Center (Centro Académico Médico de Lisboa (CAML)) and complied with national and European Union legislation for clinical research.

### Experimental procedure

The experimental session took place in a quiet room of the CAML’s Clinical Research Centre (Centro para Investigação Clínica) at the Hospital de Santa Maria, Lisbon, Portugal. We used a double-blind (throughout data collection up to statistical analysis, inclusive), randomized placebo-controlled, cross-over design, whereby each participant took part in two sessions: one for IN-OT and another for placebo administration, in a counterbalanced order, and at the same time each day (by 2 pm). The IN-OT administration of 24 IU was via three puffs of 0.1 ml each, in each nostril, from a 40 IU ml^−1^ 5 ml Syntocinon bottle (using the Novartis formula, batch H5148 produced by Huningue Production, France) or an identical placebo bottle (with the same ingredients, except OT, batch 170317.01 produced by VolksApotheke Schaffhausen, Switzerland), both supplied by Victoria Apotheke Zürich, Switzerland. 24 IU of IN-OT was used, as this dose is sufficient to increase central levels of OT to a functionally relevant degree (Quintana et al., 2021). OT and placebo sessions were approximately 7 days apart. Drug storage, administration, and drug blinding efficacy are further detailed in Supplemental Material.

**Figure 1.**
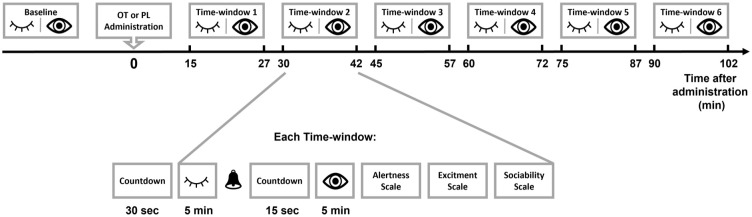
The resting-state task. Neurophysiological data were recorded in eyes-closed (HRV) and eyes-open (HRV and pupil size) conditions in seven time windows, one prior to drug administration (baseline) and six post administration. Each time window was preceded by a 30-s countdown. Then followed 5 min of eyes-closed, a beep as an instruction to open eyes, a 15-s countdown, and finally five more minutes of recording. Afterwards, the participants filled in on-screen Likert-type scales for alertness, excitement and sociability. Between time windows, participants were allowed to rest until the start of the following recording period. OT: oxytocin; PL: placebo; HRV: heart rate variability.

Participants spent seven time windows of 5-min eyes-closed and 5-min eyes-open (−10–0 min before administration and 15–27 min, 30–42 min, 45–57 min, 1 h to 1 h 12 min, 1 h 15 min to 1 h 27 min, 1 h 30 min to 1 h 42 min after administration) (see Figure 1) in which they were asked to stay still, avoid cognitive processes (e.g., mental arithmetic calculations), to relax, and, in the eyes-open condition, to fixate their gaze on a fixation cross at the center of a screen. At the end of each time window, the participants filled in three measures with Likert-type scales: alertness (1, *alert*; 5, *sleepy*), excitement (1, *excited*; 5, *calm*), and desire to socialize (1, *desire to socialize*; 5, *desire to be left alone*). EEG data were collected during the same experimental sessions and are reported elsewhere (Zelenina et al., 2022).

### Data acquisition and preprocessing

#### Pupillary activity

Participants sat comfortably with their chin supported over a chinrest to minimize head movement, at approximately 56 cm away from a Lenovo 23.8-inch screen with 1920 × 1080 resolution and 60 Hz refresh rate. At a 1000 Hz sampling rate, monocular gaze tracking and pupil size of the left eye of every participant were recorded with an SR Research EyeLink 1000 Plus which has an average accuracy of 0.15 visual angle. From the raw pupil size signal, samples 75 ms before and after blinks, as identified by the eye tracker, were converted to missing data to remove artifacts caused by partial occlusion of the eyelids (Hershman et al., 2018). Afterwards, using self-written scripts in Python v3.7.4, the signal was filtered using a third-order digital filter with a 4 Hz cutoff frequency. Missing data were linearly interpolated if it did not exceed 600 ms, as blinks longer than that are considered microsleeps (Caffier et al., 2003; Schleicher et al., 2008; Wang et al., 2011). Finally, if a 5-min time window had more than 25% of missing data, the time window was excluded from the analysis. From the fully preprocessed pupil size signal two measures were extracted, replicating Schumannet al.’s (2020) work: PUI (Lüdtke et al., 1998) and SampEn (Richman and Moorman, 2000) as each is, respectively, positively correlated to indices of PNS and SNS activity (Schumann et al., 2020). To compute the PUI, the absolute differences in the mean pupil size of consecutive segments lasting 640 ms were summed and averaged per minute (Lüdtke et al., 1998; Schumann et al., 2020). The SampEn was computed using the “pyEntropy” library (Donets et al., 2018) to input the pupil size signal down sampled to 100 Hz, embedding dimension *m* = 5 and tolerance level, *r* = 0.2 (Schumann et al., 2020).

In order to assess the possible confounding impact of the pupil foreshortening error (Hayes and Petrov, 2016) on pupil size measurements, the Euclidean distance from each sample’s location on the screen to the center (i.e., fixation cross) was subjected to the same preprocessing steps as the pupil size (see above). The main effect of drug on the Euclidean distance was not significant, *F*(1, 198.72) = 0.18, *p* = 0.671, *d* = 0.07, nor was the interaction with time, *F*(5, 191.17) = 1.12, *p* = 0.353. However, pairwise comparisons, per time window, indicated a difference in time window 2 (from 35 to 40 min), *t*(192.15) = 2.00, *p* = 0.047, *d* = 0.66, 95% confidence interval (CI) [0.01, 1.32] such that the Euclidean distance was increased under IN-OT compared to placebo.

#### Heart rate variability

The HRV was measured using a BIOPAC MP150 amplifier with the ECG recording module ECG100C-MRI in R wave at 1000 Hz sampling rate, gain as 1000, LP as 35 Hz and HP as 1 Hz (Biopac Systems Inc., Goleta, CA, USA) and AcqKnowledge 4.3 software. Three Ag/AgCl electrodes with 11-mm diameter (EL503 EKG , Biopac Systems Inc., Goleta, CA, USA) were placed in a Lead II disposition. The beat-to-beat RR intervals were analyzed using the Kubios Premium software (version 3.2) (Lipponen and Tarvainen, 2019; Tarvainen et al., 2014). A smoothness priors detrending method for trend removal was applied (delta = 500) with an interpolation rate of 4 Hz. After visual inspection and correction of missed or misaligned beats, artifact corrections were applied in 8.25% of all data with the very low (0.45 s) or low (0.35 s) thresholds. We used a piecewise cubic spline interpolation method (acceptance threshold 5%) for detecting RR intervals that were considered very different from the average RR interval for each participant (e.g., ectopic beats). To address our main research questions, we analyzed from the frequency domain the HF-HRV (frequency activity in the 0.15–0.40 Hz range), calculated using nonparametric Fast Fourier transformation absolute power (ms^2^), replicating a previous IN-OT administration at rest study (Kemp et al., 2012). As mentioned in our aims’ description, we provided in Supplemental Material the additional analysis of (1) RMSSD; (2) Kubios’ proprietary PNS and SNS indexes, the first calculated from mean RR intervals, RMSSD and Poincaré plot index S1 in normalized units, and the later calculated from mean HR, Baevky’s stress index, and Poincaré plot index S2 in normalized units; and (3) the DFAα1, particularly because it was used in a previous similar study, with statistically significant IN-OT effects (Kemp et al., 2012), albeit this measure would be more appropriately analyzed in data collected over several hours (Shaffer and Ginsberg, 2017), which we (and the previous study, in fact) have not collected.

### Statistical analysis

Statistical analysis was performed in R software 3.6 (R Core Team, 2014). A linear mixed model (LMM) was run for each dependent variable (neurophysiological data: seven in total, two for pupil size-related measures and five for HRV-related measures; behavior data: three in total one for each scale) using the package “lme4” (Bates et al., 2015) with Drug session (IN-OT, placebo), Time (post-administration time window: 1, 2, 3, 4, 5, 6) and their interaction as categorical fixed factors, and participant as a random factor. For HRV, the analysis was performed separately for each condition: eyes-open and eyes-closed. Naturally, for pupil size, the analysis was only performed for the eyes-open condition. Regarding the mood scales, we have reported on the same analysis earlier in a sample differing in one subject (Zelenina et al., 2022). LMMs are suitable for datasets with missing data and inter-individual random differences (Meteyard and Davies, 2020) and allow for the inclusion of covariates of no interest that vary within subjects. As such, herein, baseline values of each drug session (i.e., the dependent variable measured at the time window prior to drug administration: time window 0), per participant, were included in the model to account for any resulting variance. For completeness, we noted that between sessions, baseline values (i.e., before the double-blind, randomized placebo-controlled administration) were significantly higher for IN-OT than placebo for PUI (*t*(166) = 3.91, uncorrected *p* < 0.001, *d* = 0.61, 95% CI [0.29, 0.92]), SampEn (*t*(172) = 4.38, uncorrected *p* < 0.001, *d* = 0.67, 95% CI [0.36, 0.99]), and HF-HRV in the eyes-open condition (*t*(188) = 2.35, uncorrected *p* = 0.020, *d* = 0.36, 95% CI [0.06, 0.67]). There was no significant difference for HF-HRV in the eyes-closed condition (*t*(186) = 0.36, uncorrected *p* = 0.718, *d* = 0.06, 95% CI [−0.25, 0.36]). The degrees of freedom and *p*-values were calculated using type III analysis of variance with Satterthwaite’s method. We report a measure of effect size *d* for LMMs, analogue to Cohen’s *d*, for the main effect of drug (Brysbaert and Stevens, 2018), and Cohen’s *d* and 95% CI for statistically significant pairwise comparisons. We considered the main effect of drug, or of the drug-by-time interaction, on the ANS measures, to be statistically significant at a Bonferroni corrected *p*-value of 0.05 (i.e., its uncorrected *p*-values were multiplied by 3, and the resulting corrected *p*-value was thus reported, three being the number of ANS measures we analyzed). Where such a significant effect was identified, we conducted follow-up pairwise comparisons for each time window separately. These follow-up pairwise comparisons are not corrected for multiple comparisons, as that might be overly conservative given the already a priori statistical significance of the respective omnibus test (Howell, 2009; Rothman, 1990; Saville, 1990). In cases where no omnibus test was significant (both for ANS and mood data), we have refrained from highlighting or interpreting window-based pairwise comparisons even if they survived uncorrected *p* < 0.05, given the higher likelihood of false positives. Thus, these should be interpreted with caution. However, in such cases, we have reported and labelled (uncorrected) *p* < 0.05 pairwise comparisons in Results as “Exploratory,” in order to aid potential future work with a focus on temporal-dependent IN-OT effects. All pairwise comparisons were run on estimated marginal means using the EMMEANS package from R (with degrees of freedom estimated using the Kenward-Roger method which is more precise for small samples). Since the main effect of time is not relevant to our research question, we report it only in Supplemental Material and do not interpret it.

## Results

### Heart rate variability

#### Eyes-closed

**Figure 2.**
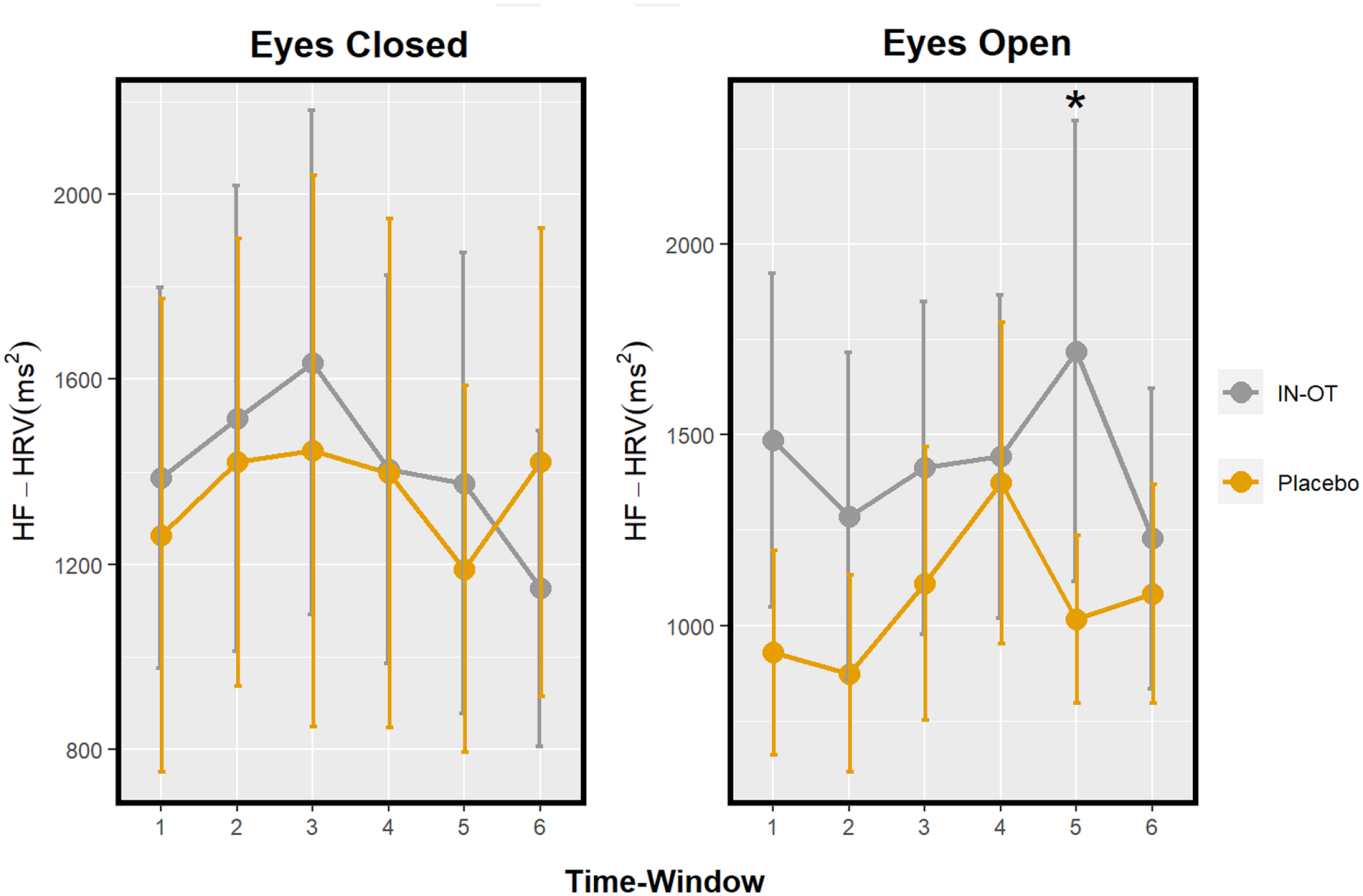
Profile of HF-HRV after IN-OT in a resting-state paradigm with eyes-closed (left) and eyes-open (right) conditions. Significant pairwise comparisons (IN-OT vs placebo) at specific time windows are marked with an asterisk (*). Eyes-closed time windows: 1 = 15–20 min; 2 = 30–35 min; 3 = 45–50 min; 4 = 60–65 min; 5 = 75–80 min; and 6 = 90–95 min. Eyes-open time windows: 1 = 20–25 min; 2 = 35–40 min; 3 = 50–55 min; 4 = 65–70 min; 5 = 80–85 min; and 6 = 95–100 min. Error bars: Standard error. HF-HRV: high-frequency heart rate variability; IN-OT: intranasal oxytocin; HRV: heart rate variability.

The main effect of drug on HF-HRV in eyes-closed, *F*(1, 184.30) = 0.18, Bonferroni corrected *p* > 0.999, *d* < 0.01, and its interaction with time, *F*(5, 171.94) = 0.22, Bonferroni corrected *p* > 0.999, was not statistically significant (Figure 2 and Table 1).

**Table 1.** Summary of the results of the effect of drug on the neurophysiological measures.

Neurophysiological measure	Main effect of drug (IN-OT vs placebo)	Pairwise comparisons per time window (if *p* < 0.05)	Drug effect direction	Tentative ANS response interpretation
Eyes-closed				
HF-HRV	*F*(1, 184.30) = 0.18, Bonferroni corrected *p* > 0.999, *d* < 0.01	–	–	–
Eyes-open				
HF-HRV	*F*(1, 175.56) = 2.11, Bonferroni corrected *p* = 0.444, *d* = 0.30	Exploratory: TW 5: *t*(173.08) = 2.28, uncorrected *p* = 0.024, *d* = 0.80, 95% CI [0.10, 1.50]	IN-OT ↑	PNS ↑
PUI	*F*(1, 167.92) = 11.42, Bonferroni corrected *p* = 0.003*, *d* = 0.45	TW 4: *t*(156.61) = 2.69, uncorrected *p* = 0.008, *d* = 0.96, 95% CI [−1.67, −0.25]	IN-OT ↓	PNS ↓
		TW 5: *t*(158.18) = 2.25, uncorrected *p* = 0.026, *d* = 0.85, 95% CI [−1.61, −0.10]	IN-OT ↓	PNS ↓
		TW 6: *t*(158.24) = 2.38, uncorrected *p* = 0.019, *d* = 0.88, 95% CI [−1.62, −0.14]	IN-OT ↓	PNS ↓
SampEn	*F*(1, 163.77) = 0.06, Bonferroni corrected *p* > 0.999, *d* = 0.54	–	–	–

All main effects of drug are shown; with statistically significant main effects (i.e., Bonferroni-corrected for the number of neurophysiological measures used) marked with an asterisk (*). Only follow-up pairwise comparisons surviving an uncorrected *p* < 0.05 are shown. Eyes-closed time windows (TWs): TW 1 =15–20 min; TW 2 = 30–35 min; TW 3 = 45–50 min; TW 4 = 60–65 min; TW 5 = 75–80 min; and TW 6 = 90–95 min. Eyes-open TWs: TW 1 = 20–25 min; TW 2 = 35–40 min; TW 3 = 50–55 min; TW 4 = 65–70 min; TW 5 = 80–85 min; and TW 6 = 95–100 min. HF-HRV: high-frequency heart rate variability; PUI: pupillary unrest index; SampEn: sample entropy; IN-OT: intranasal oxytocin; CI: confidence interval; ANS: autonomic nervous system.

#### Eyes-open

The main effect of drug on HF-HRV in eyes-open, *F*(1, 175.56) = 2.11, Bonferroni corrected *p* = 0.444, *d* = 0.30, and its interaction with time, *F*(5, 169.80) = 1.42, Bonferroni corrected *p* = 0.657, was not significant. (Exploratory pairwise comparisons in each time window indicated a significant difference in time window 5 (from 80 to 85 min), *t*(173.08) = 2.28, uncorrected *p* = 0.024, *d* = 0.80, 95% CI [0.10, 1.50], such that HF-HRV increased under IN-OT compared to placebo. (Figure 2 and Table 1).)

### Pupillary unrest (PUI and SampEn)

**Figure 3.**
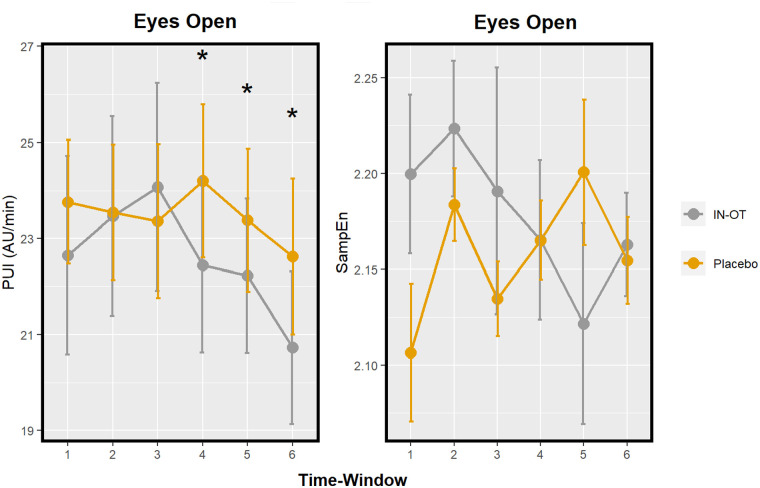
Profile of two pupil size measures after IN-OT: PUI and SampEn; in a resting-state paradigm. Significant pairwise comparisons (IN-OT vs placebo) at specific time windows are marked with an asterisk (*). Time windows: 1 = 20–25 min; 2 = 35–40 min; 3 = 50–55 min; 4 = 65–70 min; 5 = 80–85 min; and 6 = 95–100 min. Error bars: Standard error. PUI: pupillary unrest index; SampEn: sample entropy; IN-OT: intranasal oxytocin.

The main effect of drug on PUI was significant, *F*(1, 167.92) = 11.42, Bonferroni corrected *p* = 0.003, *d* = 0.45, such that PUI was decreased under IN-OT compared to placebo. Pairwise tests show this effect to be significant specifically in the last three time windows (spanning from 65 until 100 min) (respectively, *t*(156.61) = 2.69, uncorrected *p* = 0.008, *d* = 0.96, 95% CI [−1.67, −0.25]; *t*(158.18) = 2.25, uncorrected *p* = 0.026, *d* = 0.85, 95% CI [−1.61, −0.10]; *t*(158.24) = 2.38, uncorrected *p* = 0.019, *d* = 0.88, 95% CI [–1.62, –0.14] (Figure 3 and Table 1). A drug-by-time interaction on PUI was not significant, *F*(5, 155.84) = 1.60, Bonferroni corrected *p* = 0.492. The main effect of drug on SampEn, *F*(1, 163.77) = 0.06, Bonferroni corrected *p* > 0.999, *d* = 0.54, and its interaction with time were not significant, *F*(5, 154.48) = 1.72, Bonferroni corrected *p* = 0.399 (Figure 3 and Table 1).

### Behavioral and Mood scales

The main effect of drug on excitability, *F*(1, 220.37) = 0.76, Bonferroni corrected *p* > 0.999, *d* = 0.22, and its interaction with time, *F*(5, 202.49) = 1.19, Bonferroni corrected *p* = 0.939 were not significant. Exploratory pairwise comparisons revealed a significant difference between drugs in time window 5 (from 75 to 87 min), *t*(207.44) = 2.35, uncorrected *p* = 0.020, *d* = 0.76, 95% CI [0.12, 1.40], whereby excitability increased under IN-OT compared to placebo. The main effect of drug on sociability, *F*(1, 221) = 0.11, Bonferroni corrected *p* > 0.999, *d* = 0.54, and its interaction with time, *F*(5, 221) = 1.13, Bonferroni corrected *p* > 0.999, were not significant; nor on alertness, *F*(1, 206.94) < 0.01, Bonferroni corrected *p* > 0.999, *d* = 0.52; and *F*(5, 202.40) = 1.48, Bonferroni corrected *p* = 0.597, respectively.

## Discussion

In this study, we aimed, for the first time, to our knowledge, to describe the temporal profile of 24 IU of IN-OT on ANS activity at rest. We included eyes-closed and eyes-open conditions and multiple time windows across a typically large neuroscience experiment duration (including a baseline assessment prior to drug administration), where we examined two positive proxies of PNS activity (HF-HRV and PUI) (Schumann et al., 2020; Shaffer and Ginsberg, 2017) and one of SNS activity (SampEn), across two data modalities (Schumann et al., 2020). Contrary to our directional hypothesis, we found an indication that IN-OT may *deactivate* the PNS as reflected by a decrease in PUI—starting from 65-min post-administration until the end of the last window measurement (100 min). As a secondary and exploratory finding, which needs confirmation in future studies, we found a tentative indication that IN-OT may *activate* the PNS since we detected it to increase HF-HRV (only) in the 80- to 85-min post-administration window. Regarding timing, we found our peak effects of IN-OT to be later than the 40-min time window researched in most studies and lasted longer than previous IN-OT studies’ usual session length (up to 90 min) (Norman et al., 2011). Lastly, we found no significant effect of IN-OT on SampEn, thus no support for an IN-OT influence on the SNS branch when measured via pupillometry. Next, we discuss these results and offer a possible explanation for the seemingly inconsistent PNS findings (between our HRV and pupillary unrest findings) which may be due to the yet unclear reliability of the pupillary unrest markers used herein and in other studies. We noted that the following interpretations should remain tentative given the early days in IN-OT and ANS association research.

## The temporal profile of IN-OT effects

As abovementioned, the IN-OT effects we found both on pupillary unrest (at 65–100 min), and the exploratory finding on HF-HRV (at 80–85 min), were detected later than 40 min. Forty minutes is the starting time point which most previous studies have investigated (Gamer and Büchel, 2012; Kemp et al., 2012; Kubzansky et al., 2012; Leknes et al., 2013; Prehn et al., 2013; Schoormans et al., 2020; Tracy et al., 2018), with variable lengths, except one discussed below which used multiple time windows (Norman et al., 2011), and where they have mostly found significant IN-OT effects with two exceptions (Schoormans et al., 2020; Tracy et al., 2018). Those were specifically on HF-HRV at rest (Kemp et al., 2012; Kubzansky et al., 2012; Tracy et al., 2018) or on HF-HRV (Gamer and Büchel, 2012; Norman et al., 2011) and pupil dilation (Leknes et al., 2013; Prehn et al., 2013) using cognitive tasks. Nevertheless, none has explored IN-OT effects beyond 90 min or with pupillary unrest. Power differences may also explain the variable results since some have different designs (within- vs between-subject designs) and variable sample sizes (ranging from 21 to 173 subjects) (Gamer and Büchel, 2012; Kemp et al., 2012; Kubzansky et al., 2012; Leknes et al., 2013; Prehn et al., 2013; Schoormans et al., 2020; Tracy et al., 2018). Only one prior study has tested IN-OT effects in multiple time windows, as we have, albeit with cognitive tasks (Norman et al., 2011). Norman and colleagues (Norman et al., 2011) recorded autonomic cardiac indices during seven consecutive 15-min time windows, from a pre-administration baseline until 90 min post-administration of 20 UI of IN-OT. Direction-wise in line with our results, they found an IN-OT induced increase in HF-HRV as our exploratory finding herein, although theirs was in the 45–70-min time window, while ours was in the 80–85 min. We thus partially supported this finding in a resting-state paradigm. Crucially, this effect should not have been confounded by the significant difference we found in the baseline time window, in which HF-HRV was increased in the IN-OT group compared to placebo, given that we adjusted our statistical models for this by including the baseline data as a covariate of no interest (see Materials and methods’ section).

## IN-OT decreased PUI at rest: (Unexpectedly) suggestive of PNS *deactivation*?

Our finding of IN-OT having an effect on pupil size at rest, that is, a medium-sized (*d* = 0.45) main effect of drug on PUI (but large effects, *d* > 0.8, at specific time windows), was such that IN-OT, unexpectedly, *decreased* PUI from 65 min until 100 min post-administration. This was our most statistically significant finding and most novel, given the so far only indirect evidence available of PUI’s relationship with ANS function (i.e., that PUI had recently been positively associated with RMSSD, a temporal-domain cardiac positive index of PNS activity (Schumann et al., 2020)). Interestingly the same authors also found it to be associated with skin conductance indices, which in turn was found to be a positive proxy of SNS, rather than PNS, activity in healthy controls (Schumann et al., 2017). As such, what PUI is a proxy for, in terms of ANS function, is still unclear; thus, our pupillary unrest findings, although statistically significant, should remain well open to alternative interpretations.

On the other hand, and more substantially supported by previous evidence, PUI also increases with sleepiness and drowsiness and decreases with alertness (Lüdtke et al., 1998). This could be suggested to indicate that IN-OT (by decreasing PUI) increased our study participants’ vigilance and attentive state. (This was unaffected by time-of-day variations (Danker-Hopfe et al., 2001), as all recording sessions started at approximately 2.11 pm; as in Supplemental Material—Experimental Procedure.) Our mood findings did not point to an effect of IN-OT on alertness *per se*, but they did—as an exploratory finding—on the somewhat related excitability in a positive relationship. More recently, OT has been hypothesized to be associated with attention and orienting responses to external social stimuli in an interplay with the dopaminergic system (Shamay-Tsoory and Abu-Akel, 2016). As such, under the “salience hypothesis of OT,” IN-OT’s effects on PUI would not be surprising, given the association of PUI with attentive states and alertness (Lüdtke et al., 1998). The sustained increase in the attentive state under IN-OT might also be explained by the closely related “approach-withdrawal hypothesis of OT” which posits it facilitates an approach to emotionally relevant stimuli (Harari-Dahan and Bernstein, 2014). OT may serve to maintain alertness in order to promote readiness to eventually engage in approach-mediated social behaviors or readiness to eventually withdraw from social stressors (Kubzansky et al., 2012).

While PUI’s direct association with each branch of the ANS has not yet been researched, HF-HRV is one of the most robust proxies of the PNS, backed by practical and theoretical evidence (Acharya et al., 2006; Shaffer and Ginsberg, 2017). As such, although our HF-HRV significant result was found on an exploratory basis (i.e., with an increased risk of being a false positive given that the main effect of drug was not statistically significant, and the follow-up pairwise comparisons were not corrected for multiple comparisons), we herein briefly and tentatively comment on them. Our exploratory HF-HRV finding was that it increased under IN-OT at rest, in the 80- to 85-min time window, which is in the same direction as the two other IN-OT resting-state studies (Kemp et al., 2012; Kubzansky et al., 2012). Such findings suggest that IN-OT upregulates PNS and may be consistent with OT motivating approach behaviors (again, in support of the “approach-withdrawal hypothesis of OT”; Harari-Dahan and Bernstein, 2014). Alternatively, one previous study found no effect of IN-OT on HF-HRV at rest but found IN-OT to *decrease* HF-HRV during a mental arithmetic task (Tracy et al., 2018), suggesting that, in the presence of a stressor, OT inhibits the PNS rather than triggers it (causing an effect analogous to SNS activation; Chrousos and Gold, 1992), in order to solve the stressful situation and maintain an optimal internal state (Kemp et al., 2012; Kubzansky et al., 2012; Quintana et al., 2013); while, at rest, OT may induce relaxation and lowered anxiety (Dodhia et al., 2014). In sum, we speculate that although our HF-HRV exploratory finding seems to contradict our PUI finding, the former has directional support from previous IN-OT studies (Kemp et al., 2012; Kubzansky et al., 2012), and both might be consistent with the facilitation of alertness and preparedness for an approach behavior, given previous evidence (Baethge et al., 2019; Beffara et al., 2016; Harari-Dahan and Bernstein, 2014; Kemp et al., 2012).

## Limitations

Herein we computed the Euclidean distance from each sample’s location to the center of the screen (i.e., fixation cross) and subjected this measure to the same statistical analysis of our dependent variables—to assess a, by chance, possible confounding effect of the pupil foreshortening error on our drug effect analyses (Hayes and Petrov, 2016). This was not verified, as this measure was (positively) associated with the drug effect only in the 35- to 40-min time window (eyes-open time window 2), where we report no statistically significant effects. Additionally, we recognize variable IN-OT dosages would have allowed us to improve our pharmacokinetic modeling; nevertheless, we chose the most commonly administered dosage in the literature for comparability (Zelenina et al., 2022). Although an apparent limitation, we have also not measured OT blood levels as (i) they do not necessarily reflect CNS activity and (ii) they could represent the simulation of endogenous OT release, as well as the administered OT (Martins et al., 2020) and (iii) the stress-inducing phlebotomy has noisy effects on ANS activity (Alley et al., 2019; Brown et al., 2016). We also reiterate that our choice of conducting an exploratory analysis of this dataset and thereby presenting follow-up pairwise tests without correction for multiple comparisons (even when the omnibus test was not significant) means that pairwise tests relating to HF-HRV carry an increased risk of false positivity and therefore should be interpreted with caution. In addition, power limitations may have prevented the detection of significant IN-OT effects on HF-HRV consistently across all time windows, as others have achieved with a sample size doubling ours (Norman et al., 2011). However, while the latter employed a between-subjects design, our within-subject design should have been equally powerful with a smaller sample. Indeed, like us, another study reported the effects of IN-OT on HF-HRV with approximately 20 subjects in a within-subject design (Kemp et al., 2012), while another with an increased sample size (IN-OT *N* = 87 and placebo *N* = 86) in a between-subject design, found no such effects (Schoormans et al., 2020). Overall, given the mixed literature and the early days of ANS and IN-OT research, we cannot so far exclude that the measures we used are not robust markers of IN-OT effects. Finally, our results reflect the temporal profile of IN-OT on male ANS activity and cannot be generalized to females, given evidence that OT baseline levels are 3× higher in women than in men, as measured in plasma (Marazziti et al., 2019), that menstrual cycle impacts OT levels (Mitchell et al., 1981; Salonia et al., 2005; Stock et al., 1991), and of sex-related differences in several functional effects of IN-OT (Evans et al., 2014). The same evidence was behind our choice of restricting our target population to males to increase statistical power and reduce model complexity.

## Conclusions

We report herein on the temporal profile of IN-OT in the human ANS at rest using HRV and, for the first time, pupillary unrest measures. Having found OT to decrease PUI (suggesting PNS deactivation) and tentatively (as an exploratory finding) that it may increase HF-HRV (suggesting PNS activation), we speculated that both might be consistent with the facilitation of alertness and preparedness for an approach behavior. Given the early days of IN-OT and ANS association research, the interpretation of these results remains speculative. Nevertheless, we hope our findings may assist in future study design, in the investigation of the comparability between IN-OT findings across data modalities, and in the assessment of usefulness of ANS markers for IN-OT response monitoring and human social cognition understanding.

## Supplemental Material

sj-docx-1-jop-10.1177_02698811231158233 – Supplemental material for Temporal profile of intranasal oxytocin in the human autonomic nervous system at rest: An electrocardiography and pupillometry study Click here for additional data file. Supplemental material, sj-docx-1-jop-10.1177_02698811231158233 for Temporal profile of intranasal oxytocin in the human autonomic nervous system at rest: An electrocardiography and pupillometry study by Gonçalo Cosme, Patrícia Arriaga, Pedro J. Rosa, Mitul A. Mehta and Diana Prata in Journal of Psychopharmacology
