# Supplementary material for: Temporal profile of intranasal oxytocin in the human autonomic nervous system at rest: An electrocardiography and pupillometry study
Source: J Psychopharmacol. 2023 Mar 9;37(6):566–76. doi: 10.1177/02698811231158233 (PMC10291383; doi:10.1177/02698811231158233)
Supplement: sj-docx-1-jop-10.1177_02698811231158233 – Supplemental material for Temporal profile of intranasal oxytocin in the human autonomic nervous system at rest: An electrocardiography and pupillometry study [file sj-docx-1-jop-10.1177_02698811231158233.docx]

Supplementary Material for:

# **Temporal profile of intranasal oxytocin in the human autonomic nervous system at rest: an electrocardiography and pupillometry study**

Gonçalo Cosme^1^, MSc, Patrícia Arriaga^2^, PhD, Pedro J. Rosa^,3,4^, PhD, Mehta MA^5^, PhD, Diana Prata^1,5^, PhD

**Corresponding author**: Dr. Diana Prata, Instituto de Biofísica e Engenharia Biomédica, Faculdade de Ciências da Universidade de Lisboa, Campo Grande 016, 1749-016 Lisboa, Portugal; +351 21 750 0177; diana.prata@kcl.ac.uk

**Supplementary details for Materials and Methods:**

**Experimental procedure:** Drug acquisition, storage and randomization of drug administration was performed and controlled by the hospital’s pharmacy. IN-OT/placebo administration was at 2:24 pm (SD = 29 min) for all participants, and HRV and pupil recording at 2:11 pm, to restrict the impact of the circadian rhythm on baseline endogenous OT levels (Forsling, 2000; Forsling et al., 1993). Screening for eligibility was performed via self-report during an initial phone interview and in person via questionnaire, upon arrival to the first session, and their health state was assessed via medical examination, which included heart rate, blood pressure and electrocardiogram measurements. In the second session, only the eligibility questionnaire was administered.

To confirm the drug blinding procedure we asked the participants at the end of Session 2 to guess in which session they would have self-administered IN-OT. Not only were they unable to do so correctly, above chance-level; the frequency of correct guesses did not differ significantly between drug groups, within each session. In Session 1, 37.5% guessed IN-OT while being administered IN-OT, compared to 70% guessing IN-OT while being administered placebo (*p* = .184, Fisher’s exact test); in Session 2, 30% guessed IN-OT while being administered IN-OT, compared to 75% guessing IN-OT while being administered placebo (*p* = .077, Fisher’s exact test).

**Supplementary Results:**

**Results – Kubio’s proprietary PNS and SNS indexes, DFAα1 and RMSSD**

***Eyes closed.*** The exploratory analysis of the main effect of drug on Kubios’ PNS index, *F*(1, 176.68) = 3.22, *p* = .075, *d* = 0.21, and its interaction with time, *F*(5, 169.11) = 0.31, *p* = .909 were not significant.

The exploratory analysis of the main effect of drug on Kubios’ SNS index was statistically significant, *F*(1, 175.47) = 4.94, *p* = .028, *d* = 0.32, indicating that the SNS index increased under IN-OT compared to placebo. The interaction with time was not significant, *F*(5, 169.32) = 0.64, *p* = .668.

The main effect of drug on DFAα1 *F*(1, 182.77) = 0.73, *p* = .396, *d* = 0.08 and its interaction with time, *F*(5, 165.95) = 1.18, *p* = .321, were not significant. However, pairwise comparisons indicated a significant difference between conditions in the last time-window (from 90 to 95 min), *t*(175) = 2.33, *p* = .021, *d* = 0.81, 95% CI [0.12, 1.51], such that DFAα1 increased under IN-OT compared to placebo.

Lastly, the main effect of drug on RMSSD, *F*(1, 177.67) = 2.92, *p* = .089, *d* = 0.19 and its interaction with time, *F*(5, 169.39) = 0.29, *p* = .920, were not significant.

***Eyes open.*** The exploratory analysis of the main effect of drug on Kubios’ PNS index *F*(1, 172.63) = 1.13, *p* = .288, *d* = 0.39, and its interaction with time *F*(5, 162.83) = 0.68, *p* = .641, were not significant.

The exploratory analysis of the main effect of drug on Kubios’ SNS index *F*(1, 176.04) = 0.04, *p* = .845, *d* = 0.43, and its interaction with time *F*(5, 166.78) = 1.17, *p* = .327 revealed it was not significant, but exploratory pairwise comparisons indicated a difference in the first time-window (from 20 to 25 minutes) *t*(173.61) = 1.98, *p* = .049, *d* = 0.69, 95% CI [-1.39, 0.00], such that Kubios’ SNS index decreased under IN-OT compared to placebo.

The main effect of drug on DFAα1 *F*(1, 183.25) = 3.07, *p* = .081, *d* = 0.43, and its interaction with time *F*(5, 171.04) = 0.54, *p* = .749, was not significant.

Lastly, the main effect of drug on RMSSD *F*(1, 173.66) = 0.76, *p* = .385, *d* = 0.38, and its interaction with time *F*(5, 164,57) = 0.862, *p* = .508 were not significant. However, exploratory pairwise comparisons indicated a difference in the first time-window (from 20 to 25 minutes) *t*(173.49) = 1.99, *p* = .048, such that RMSSD was increased under IN-OT compared to placebo.

**Supplementary Table 1 –** Summary of the results of the effect of drug on Kubio’s proprietary PNS and SNS indexes, DFAα1 and RMSSD. Statistically significant (uncorrected *p* < .05) main effects are marked with and asterisk (*) and only follow-up pairwise comparisons surviving an uncorrected *p* < .05 are shown .

| **Neurophysiological Measure** | **All main effects of drug**  **(IN-OT vs placebo)** | **Pairwise comparisons per TW (if p< .05)** | **Drug effect direction** | **Tentative ANS response interpretation** |
| --- | --- | --- | --- | --- |
| **Eyes Closed** | | | | |
| **Kubio’s PNS index** | *F*(1, 176.68) = 3.22, *p* = .075, *d* = 0.21 | - | - | - |
| **Kubio’s SNS index** | *F*(1, 175.47) = 4.94, *p* = .028*, *d* = 0.32 | - | IN-OT ↑ | SNS ↑ |
| **DFAα1** | *F*(1, 182.77) = 0.73, *p* = .396, *d* = 0.08 | TW 6 - *t*(175) = 2.33, *p* = .021,  *d* = 0.81, 95% CI [0.12, 1.51] | IN-OT ↑ | PNS ↓  SNS ↓ |
| **RMSSD** | *F*(1, 177.67) = 2.92, *p* = .089, *d* = 0.19 | - | - | - |
| **Eyes Open** | | | | |
| **Kubio’s PNS index** | *F*(1 172.63) = 1.13, *p* = .288, *d* = 0.39 | - | - | - |
| **Kubio’s SNS index** | *F*(1 176.04) = 0.04, *p* = .845, *d* = 0.43 | TW 1 - *t*(173.61) = 1.98, *p* = .049,  *d* = 0.69, 95% CI [-1.39, 0.00] | IN-OT ↓ | SNS ↓ |
| **DFAα1** | *F*(1, 183.25) = 3.07, *p* = .081, *d* = 0.43 | - | - | - |
| **RMSSD** | *F*(1, 173.66) = 0.76, *p* = .385, *d* = 0.38 | TW 1 – *t*(173.49) = 1.99, *p* = .048,  *d* = 0.70, 95% CI [0.00, 1.40] | IN-OT ↑ | PNS ↑ |

**Footnote:** Eyes closed time-windows (TWs): 1 =15 – 20 min; 2 = 30 – 35 min; 3 = 45 – 50 min; 4 = 60 – 65 min; 5 = 75 – 80 min; and 6 = 90 – 95 min. Eyes open TWs: 1 = 20 – 25 min; 2 = 35 – 40 min; 3 = 50 – 55 min; 4 = 65 – 70 min; 5 = 80 – 85 min; and 6 = 95 – 100 min., TW = time-window, PNS = parasympathetic nervous system, SNS = sympathetic nervous system, DFAα1 = detrended fluctuation analysis scaling exponent, RMSSD = root mean square of successive differences, IN-OT = intranasal oxytocin, CI = confidence interval.


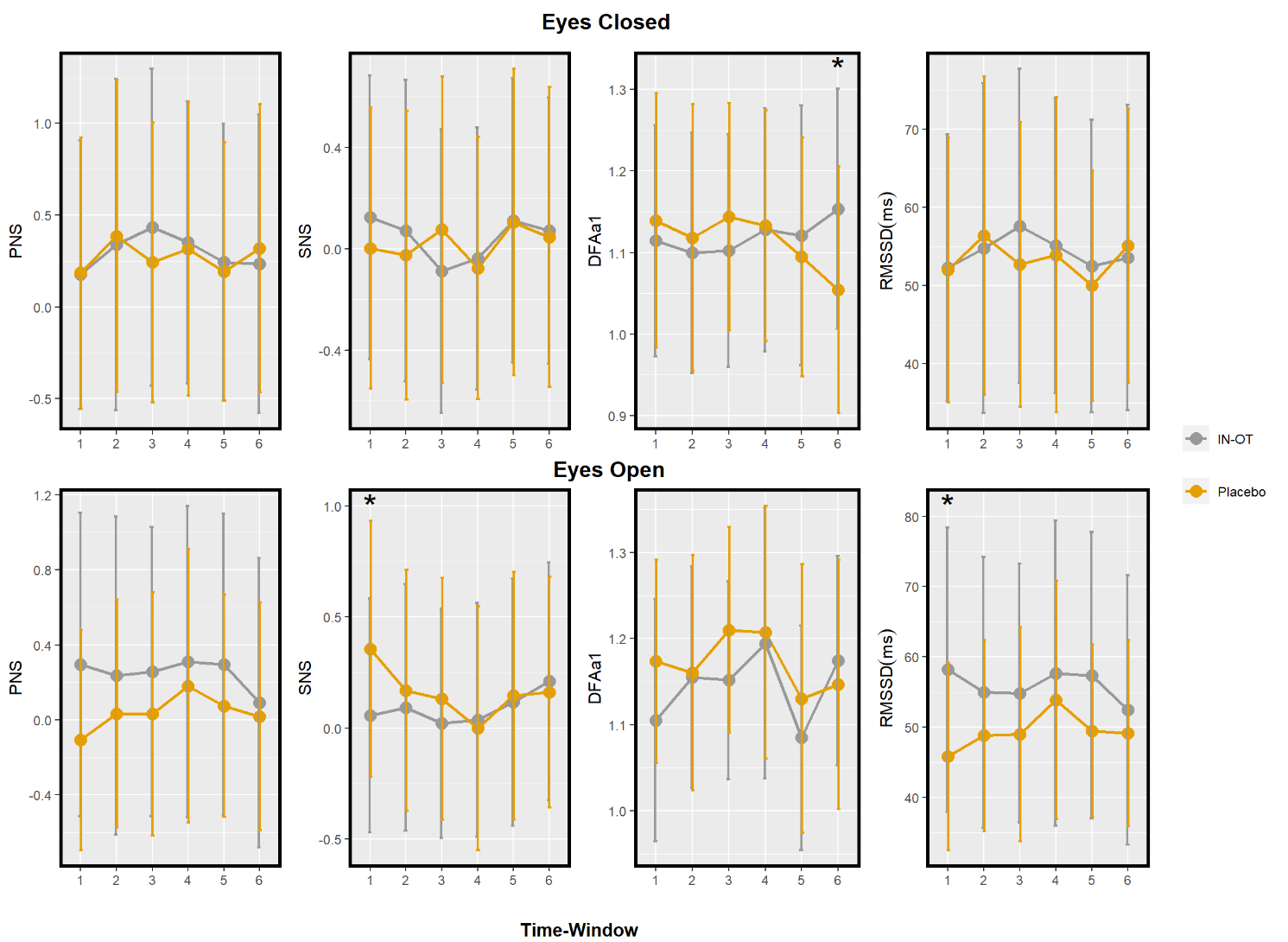


**Supplementary Figure 1 –** Profile of 4 HRV measures after IN-OT: Kubio’s proprietary PNS index (1st column) and SNS index (2nd column), DFAα1 (3rd column) and RMSSD (4th column); in a resting-state paradigm with eyes closed (top row) and eyes open (bottom row) conditions. A significant pairwise comparison (IN-OT vs placebo) at specific time-windows are marked with an *. Eyes closed condition: time-window 1 =15 – 20 min; time-window 2 = 30 – 35 min; time-window 3 = 45 – 50 min; time-window 4 = 60 – 65 min; time-window 5 = 75 – 80 min; and time-window 6 = 90 – 95 min. Eyes open condition: time-window 1 = 20 – 25 min; time-window 2 = 35 – 40 min; time-window 3 = 50 – 55 min; time-window 4 = 65 – 70 min; time-window 5 = 80 – 85 min; and time-window 6 = 95 – 100 min. Error bars: Standard error. HF-HRV = high frequency heart rate variability, IN-OT = intranasal oxytocin, HRV = heart rate variability, PNS = parasympathetic nervous system, SNS = sympathetic nervous system, DFAα1 = detrended fluctuation analysis scaling exponent, RMSSD = root mean square of successive differences.

**Results – Main effect of time**

***Eyes closed.*** The main effect of time on all measure of HRV was not statistically significant: HF-HRV *F*(5, 171.94) = 0.32, *p* = .901; Kubio’s proprietary PNS index *F*(5, 169.11) = 0.71, *p* = .620; Kubio’s proprietary SNS index *F*(5, 169,32) = 0.87, *p* = .506; DFAα1 *F*(5, 165,95) = 0.23, *p* = .948; and RMSSD *F*(5, 169,39) = 0.56, *p* = .729*.*

***Eyes open.*** The main effect of time on PUI was statistically significant *F*(5, 156.00) = 2.33, *p* = .045 but not on SampEn *F*(5, 154.59) = 0.77, *p* = .571.

The main effect of time on all measures of HRV was not statistically significant: HF-HRV *F*(5, 169.80) = 1.52, *p* = .185; Kubio’s proprietary PNS index *F*(5, 162.83) = 0.98, *p* = .434; Kubio’s proprietary SNS index *F*(5, 166,78) = 1.39, *p* = .231; DFAα1 *F*(5, 171,04) = 1.69, *p* = .139; and RMSSD *F*(5, 164,57) = 0.98, *p* = .433.

***Behavioural - Mood Scales.*** The main effect of time was not statistically significant in any of the mood scales: alertness *F*(5, 202.40) = 0.70, *p* = .622; arousal *F*(5, 202.49) = 0.14, *p* = .984; and sociability *F*(5, 221) = 0.48, *p* = .791.

**References**

Forsling ML (2000) Diurnal rhythms in neurohypophysial function. *Experimental Physiology* 85 S(SPEC. ISS.). John Wiley & Sons, Ltd: 179s–186s. DOI: 10.1111/j.1469-445x.2000.tb00022.x.

Forsling ML, Stoughton RP, Zhou V, et al. (1993) The role of the pineal in the control of the daily patterns of neurohypophysial hormone secretion. *Journal of Pineal Research* 14(1). John Wiley & Sons, Ltd: 45–51. DOI: 10.1111/j.1600-079X.1993.tb00484.x.
